# Early-pregnancy HDL-related inflammatory indices and risk of preeclampsia: A retrospective cohort study

**DOI:** 10.1371/journal.pone.0339322

**Published:** 2025-12-30

**Authors:** Hui Zhang, Jin-Lan Bao, Qian-Nan Li, Kun-Duo Xiao, Jin-Qiu Peng

**Affiliations:** Department of Clinical Laboratory, Huidong County Maternal and Child Health Hospital, Sichuang, China; Mazandaran University of Medical Sciences, IRAN, ISLAMIC REPUBLIC OF

## Abstract

**Background:**

Preeclampsia (PE) is closely associated with dyslipidemia and inflammatory imbalance. High-density lipoprotein (HDL)-related inflammatory indices have demonstrated predictive potential in cardiovascular and metabolic diseases, but evidence regarding their role in PE remains limited.

**Objective:**

This study aimed to investigate the associations between early-pregnancy HDL-related inflammatory indices, including the lymphocyte-to-HDL ratio (LHR), monocyte-to-HDL ratio (MHR), neutrophil-to-HDL ratio (NHR), and platelet-to-HDL ratio (PHR), and the risk of PE.

**Methods:**

A total of 5,959 women with singleton pregnancies were retrospectively included. All participants underwent complete blood counts (CBC) and lipid profiling at 11–13 weeks of gestation, from which HDL-related inflammatory indices were calculated. Associations between these indices and PE risk were systematically evaluated using modified Poisson regression, restricted cubic splines (RCS), subgroup analyses, and sensitivity analyses. To assess their potential clinical utility, prediction models incorporating HDL-related inflammatory indices were developed and evaluated using receiver operating characteristic (ROC) curves, calibration plots, and decision curve analysis (DCA).

**Results:**

Among 5,959 participants, 124 (2.08%) developed PE. Multivariable-adjusted analyses revealed that higher levels of LHR (RR = 1.99, 95% CI: 1.17–3.42, *P* = 0.012), MHR (RR = 2.62, 95% CI: 1.58–4.35, *P* < 0.001), NHR (RR = 2.87, 95% CI: 1.64–5.02, *P* < 0.001), and PHR (RR = 2.57, 95% CI: 1.48–4.48, *P* < 0.001) were significantly associated with increased PE risk. Quartile analyses demonstrated a dose-response relationship across categories (*P* for trend < 0.05). RCS revealed U-shaped associations for LHR (*P* = 0.008) and NHR (*P* = 0.048), which became approximately linear after excluding extreme values. Subgroup analyses suggested stronger associations in women with gestational diabetes mellitus (GDM), advanced maternal age, or higher BMI, indicating potential effect modification. Sensitivity analyses confirmed the consistency of these associations. Incorporation of these indices modestly improved model discrimination (AUC 0.697) and provided limited clinical benefit in DCA.

**Conclusions:**

Early-pregnancy HDL-related inflammatory indices, particularly MHR, NHR, and PHR, are significantly associated with increased PE risk. These indices reflect both lipid metabolism and inflammatory status, offering simple, accessible biomarkers for early risk stratification and potential supplementation of existing PE prediction strategies.

## Introduction

PE is a pregnancy-specific hypertensive disorder and remains a major cause of maternal and neonatal morbidity and mortality worldwide [1–3]. In addition to hypertension and proteinuria, PE is often accompanied by dyslipidemia and immune-inflammatory imbalance [4–6]. These alterations suggest that interactions between metabolic and immune pathways may play an important role in its pathogenesis and progression.

Dyslipidemia plays a particularly important pathological role in PE. Multiple studies have reported that High-density lipoprotein cholesterol (HDL-C) levels are significantly reduced in PE patients [7–9]. HDL-C not only transports lipids but also exerts anti-inflammatory, antioxidant, and vasculoprotective effects; its reduction, often linked to abnormal lipid metabolism, may contribute to placental dysfunction, endothelial injury, and exacerbated systemic inflammation via activation of the monocyte–macrophage system and pro-inflammatory cytokine release [7,10–12]. Moreover, the pathological features of PE share similarities with atherosclerosis, including endothelial dysfunction, chronic inflammation, and lipid metabolic disorders [13]. This similarity further supports potential crosstalk between lipid metabolism and inflammatory pathways [13].

Peripheral inflammatory markers also play an important role in the development and progression of PE. Studies have shown that PE patients exhibit elevated and aberrantly activated neutrophils in peripheral blood, with cell counts positively correlated with disease severity [14–18]. Total CD14 ⁺ monocyte proportions are increased, particularly the intermediate monocyte subset [19,20]. Platelet activation or elevated mean platelet volume (MPV) in early pregnancy may predict PE onset [21,22]. Increased lymphocyte counts have also been associated with higher PE risk [23]. These findings suggest that lipids and immune cells may jointly contribute to PE pathogenesis, while single lipid or inflammatory markers alone may not fully capture the complex pathological state.

Several predictive tools have been developed for early screening of PE, including angiogenic biomarkers such as sFlt-1/PlGF, mean arterial pressure (MAP), and uterine artery Doppler indices, among others [24,25]. However, some of these approaches are affected by factors such as gestational timing, testing platform availability, and predictive specificity, which may limit their widespread use in routine clinical practice [15,26]. Therefore, increasing research has focused on identifying simple, easily accessible blood-based biomarkers that may serve as useful adjuncts to existing screening strategies.

Based on this rationale, biomarkers that simultaneously reflect immune-inflammatory activity and lipid regulation may provide stronger biological relevance and enhanced predictive performance compared with single-dimension indicators. In this context, HDL-related inflammatory markers have emerged as promising adjuncts that could supplement current PE risk assessment models. To better capture the interaction between inflammation and lipid metabolism, researchers have proposed HDL-related inflammatory indices, including the LHR, MHR, NHR, and PHR [27,28]. These indices integrate the activation status of peripheral inflammatory cells with the protective function of HDL-C, reflecting both enhanced pro-inflammatory states and impaired vascular protection. Previous studies have demonstrated that LHR, MHR, NHR, and PHR, as integrative markers of peripheral inflammation and HDL-C function, have predictive value in cardiovascular diseases, metabolic syndrome, and certain high-risk populations [29–33].

However, systematic evaluation of HDL-related inflammatory indices (LHR, MHR, NHR, and PHR) in PE remains limited, particularly in large, ethnically specific cohorts and during early pregnancy. Given their accessibility and low cost, these indices may serve as useful adjuncts to existing prediction tools and hold potential for early risk stratification. Therefore, in a large retrospective Chinese cohort, we investigated the association between these early-pregnancy indices and subsequent risk of PE, with the aim of assessing their utility as supportive markers for early risk stratification and potential guidance for prenatal monitoring or preventive strategies.

## Materials and methods

### Study population

**Fig 1:**
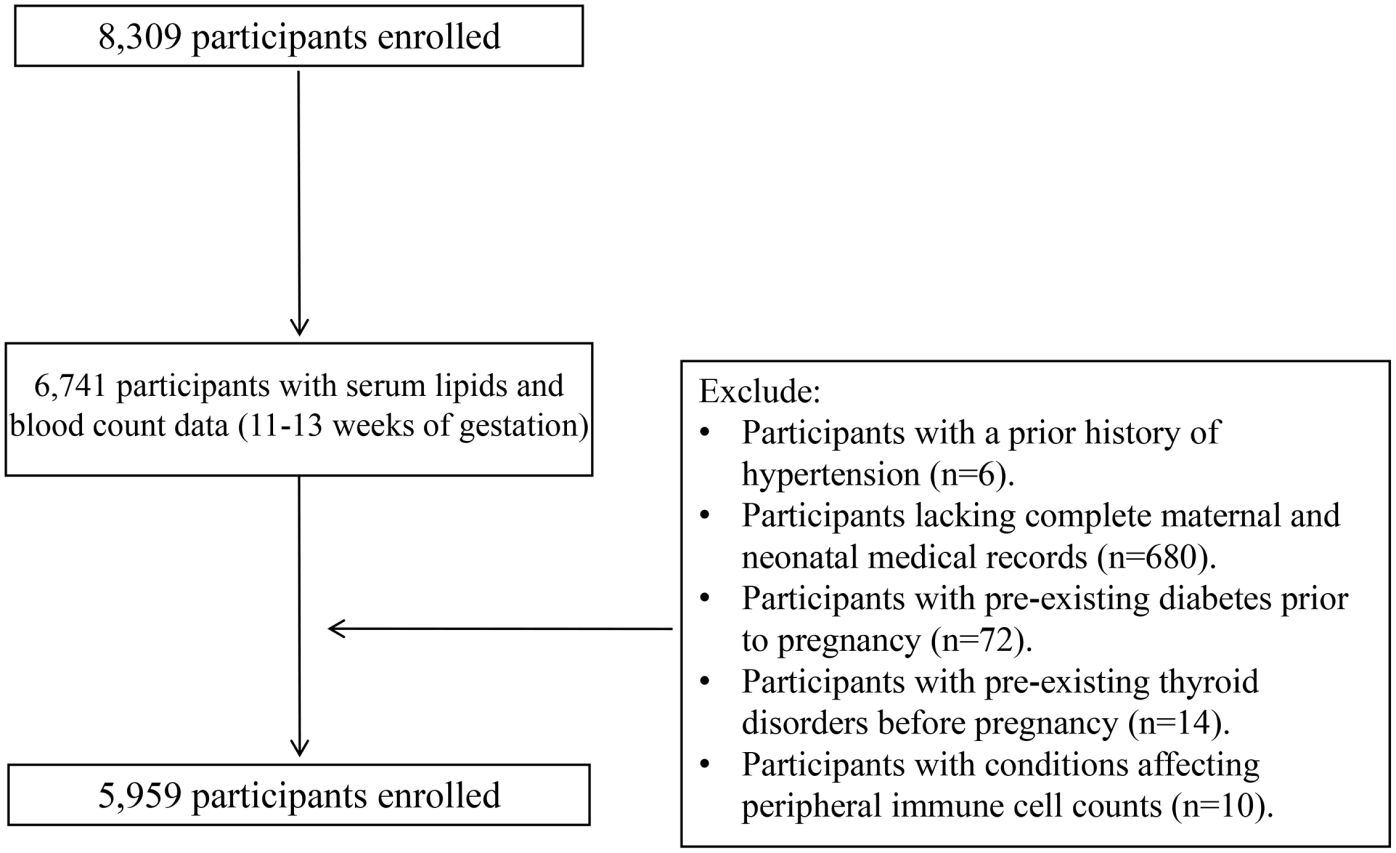
Participant inclusion and exclusion flowchart.

This study included women with singleton pregnancies who received routine prenatal care and delivered at Huidong County Maternal and Child Health Hospital between January 1, 2020 and June 30, 2025 (study period). All participants were followed until delivery. Clinical and laboratory data were extracted from the hospital information system between July 1 and July 10, 2025, and the final analytical dataset was locked on July 10, 2025 (data freeze date). Eligible participants had complete first-trimester (11–13 weeks’ gestation) blood count and lipid profile results for calculation of HDL-related inflammatory indices (LHR, MHR, NHR, and PHR) and complete maternal and neonatal medical records. Women were excluded if they had pre-existing hypertension, diabetes, or thyroid disorders, multiple pregnancies, or conditions potentially affecting peripheral immune cell counts (including hematological, autoimmune, chronic inflammatory diseases, or malignancies), or if laboratory or clinical data were missing or incomplete. In addition, women diagnosed with early-onset PE (< 34 gestational weeks) were excluded. Early- and late-onset PE are increasingly recognized as clinically and biologically distinct subtypes, conventionally differentiated by gestational age at onset. Early-onset PE is more strongly associated with abnormal placentation and fetal growth restriction, whereas late-onset PE is more commonly linked to maternal metabolic and inflammatory factors. Given these differences and the study objective, analyses were restricted to late-onset PE [34]. A total of 5,959 participants met the inclusion criteria and were included in the study (Fig 1).

### Variables

Baseline demographic and clinical characteristics, including maternal age, pre‐pregnancy body mass index (BMI), gestational weight gain, parity, gravidity, fasting glucose, and pregnancy complications, were obtained from electronic medical records.

### Measurement of HDL-related inflammatory indices

The exposures in this study were four HDL-related inflammatory indices: the LHR, MHR, NHR, and PHR. These indices were calculated using the following formulas:


$$LHR\;=\;Lymphocyte\;count\;(\text{1}{\text{0}}^{9}/L)\;/\;HDL\text{-}C\;(mmol/L))$$

$$MHR\;=\;Monocyte\;count\;(\text{1}{\text{0}}^{9}/L)\;/\;HDL\text{-}C\;(mmol/L)$$

$$NHR\;=\;Neutrophil\;count\;(\text{1}{\text{0}}^{9}/L)\;/\;HDL\text{-}C\;(mmol/L)\mathrm{\backslash ]}$$

$$PHR\;=\;Platelet\;count\;(\text{1}{\text{0}}^{9}/L)\;/\;HDL\text{-}C\;(mmol/L)\mathrm{\backslash ]}$$

Exposure data were obtained from the first CBC and lipid profile collected between 11 and 13 gestational weeks. All blood samples were collected in the morning after an 8-hour overnight fast. Venous blood was drawn from the antecubital vein using EDTA tubes for CBC and serum separator tubes for lipid measurements, and all samples were analyzed within 24 hours in the hospital’s central laboratory. Whole blood for CBC was analyzed directly without centrifugation, while serum for lipid measurements was obtained after centrifugation at 3000 × g for 10 minutes at room temperature. CBC was measured using the Mindray BC-5390CRP analyzer, and lipid profiles were measured on the Mindray BS-600 platform. Internal quality control was performed daily using control samples at multiple concentration levels. In accordance with WS/T 403–2024 and WS/T 406–2024 standards, within-run coefficients of variation were < 3% for white blood cells, < 6% for platelets, and < 6% for HDL-C. All laboratory results were systematically recorded in the hospital’s electronic medical record system for data extraction.

### Definition and ascertainment of PE

The primary outcome of this study was the occurrence of late-onset PE, defined as PE diagnosed at or after 34 weeks of gestation [35]. The diagnosis of PE was established in strict accordance with the clinical criteria of the American College of Obstetricians and Gynecologists (ACOG) guidelines, defined as new-onset hypertension after 20 weeks of gestation (systolic blood pressure ≥140 mmHg and/or diastolic blood pressure ≥90 mmHg, measured on at least two occasions 4 hours apart), accompanied by proteinuria (24-hour urinary protein ≥300 mg or a protein-to-creatinine ratio ≥0.3) or evidence of end-organ dysfunction (including thrombocytopenia, impaired liver function, renal insufficiency, pulmonary edema, or new-onset cerebral or visual disturbances) [36]. All cases were manually validated by reviewing electronic medical records, including clinical notes, laboratory results, and diagnostic reports, to ensure diagnostic accuracy.

### Statistical analyses

#### Comparison of baseline characteristics.

Baseline demographic and clinical characteristics were compared between women with and without PE. Continuous variables with approximately normal distributions were expressed as mean ± standard deviation and compared using Student’s t-test; non-normally distributed variables were summarized as median (interquartile range) and compared using the Mann-Whitney U test. Categorical variables were presented as numbers (percentages) and compared using the chi-square test or Fisher’s exact test, as appropriate.

#### Assessment of associations using modified poisson regression.

The associations between HDL-related inflammatory indices (LHR, MHR, NHR, PHR) and the risk of PE were evaluated using modified Poisson regression with robust variance estimators to estimate relative risks (RR) and 95% confidence intervals (CI). Each index was analyzed in two forms: (1) as a continuous variable (log-transformed to improve normality), and (2) as a categorical variable based on quartiles. Trends across quartiles were tested by assigning the median value to each category and treating it as a continuous variable.

#### Evaluation of non-linearity using RCS.

RCS models were applied to assess potential non-linear dose–response relationships between each inflammatory index and PE risk. Knot locations were selected using Akaike Information Criterion (AIC), and the model with the lowest AIC was retained. A sensitivity analysis excluding observations below the 1st percentile and above the 99th percentile was performed to evaluate robustness. Non-linearity was assessed using likelihood ratio tests.

### Subgroup and interaction analyses

Stratified analyses were performed by maternal age (<35 vs. ≥ 35 years), pre-pregnancy BMI (<24 vs. ≥ 24 kg/m²), and GDM status. Multiplicative interactions were evaluated by incorporating cross-product terms into the regression models and assessing their significance using the Wald test. Additive interactions were quantified using the relative excess risk due to interaction (RERI) and the attributable proportion due to interaction (AP), along with their 95% CI, calculated via the delta method.

### Sensitivity analyses

To assess the robustness of the primary findings, the following sensitivity analyses were conducted:

(1) Associations were re-estimated using multivariable logistic regression models, with results expressed as odds ratios (ORs) and 95% CIs;

(2) Propensity score matching (PSM) at a 1:4 ratio was performed using maternal age, gestational weight gain, pre-pregnancy BMI, GDM, fasting blood glucose, gestational age at blood testing, and parity as matching variables. Modified Poisson regression was then applied to the matched cohort to reassess the associations.

### Prediction modeling and performance assessment

To evaluate the predictive performance of HDL-related inflammation indices for incident PE, multiple prediction models were constructed and compared:

Base model: included established clinical risk factors, namely maternal age, pre-pregnancy BMI, gestational weight gain, GDM status, fasting glucose, gestational age at blood sampling, and parity.

Single-index models: the log-transformed LHR, MHR, NHR, or PHR was individually added to the base model.

All-exposure model: all four HDL-related inflammation indices were simultaneously added to the base model.

All models were fitted using modified Poisson regression. Discrimination was quantified using the area under the ROC curve (AUC) with corresponding 95% CIs, and between-model differences in AUC were compared using the DeLong test. Calibration was assessed using the calibration intercept and slope. DCA was performed to evaluate the net clinical benefit of each model across a range of threshold probabilities.

### Covariate selection

Covariates in all multivariable models were selected a priori based on clinical relevance and previous literature. Adjusted models included maternal age, gestational weight gain, pre-pregnancy BMI, GDM, fasting blood glucose, gestational age at the time of blood testing, and parity [7,37–42]. Multicollinearity was assessed using variance inflation factors (VIF), with a VIF < 5 considered acceptable [43].

All statistical analyses were conducted using R software (version 4.3.1), with a two-sided significance threshold of *P* < 0.05 unless otherwise specified.

### Ethical approval

The study protocol was reviewed and approved by the Ethics Committee of Huidong County Maternal and Child Health Hospital (Initial Approval No. (2019) Medical Ethics Review No. (2); Continuation Approval No. (2024) Medical Ethics Review No. (3)). All research procedures complied with the relevant ethical standards and regulations. Written informed consent was obtained from each participant after they received detailed information regarding the study.

## Result

### Baseline characteristics

A total of 5,959 women met the inclusion criteria, among whom 124 (2.08%) developed PE (Table 1). Compared with women with normal blood pressure, those with PE were older and had higher pre-pregnancy BMI, fasting glucose levels, the prevalence of GDM, and cesarean section rates. HDL-related inflammation indices, including LHR, MHR, NHR, and PHR, were significantly elevated in women with PE, whereas HDL-C levels were lower (all *P* < 0.05).

**Table 1:** Baseline characteristics by preeclampsia (PE) status.

Variables	Total	Non-PE	PE	*P*
	(n = 5,959)	(n = 5,835)	(n = 124)	
^a^Age (years)	30.34 ± 3.62	30.31 ± 3.61	31.64 ± 3.91	<.001
^a^Pre-pregnancy weight (kg)	53.93 ± 6.94	53.88 ± 6.92	56.43 ± 7.42	<.001
^a^Gestational weight gain (kg)	13.34 ± 3.93	13.34 ± 3.92	13.37 ± 3.99	0.935
Pre-pregnancy BMI (kg/m2)	21.07 ± 2.55	21.05 ± 2.54	22.31 ± 2.76	<.001
Gravidity	2.00 (1.00, 3.00)	2.00 (1.00, 3.00)	2.00 (1.00, 3.00)	0.901
Parity	1.00 (1.00, 2.00)	1.00 (1.00, 2.00)	1.00 (1.00, 2.00)	0.360
^a^Fasting blood glucose	4.61 (4.47, 4.79)	4.61 (4.46, 4.79)	4.72 (4.52, 4.88)	<.001
Gestational age at the time of blood testing	12.29 (12.00, 12.71)	12.29 (12.00, 12.71)	12.36 (11.96, 12.71)	0.681
WBC, 10^9^/L	7.78 (6.70, 9.04)	7.77 (6.69, 9.02)	8.48 (7.32, 9.99)	<.001
Monocytes, 10^9^/L	0.35 (0.29, 0.43)	0.35 (0.29, 0.43)	0.40 (0.30, 0.47)	<.001
Lymphocytes, 10^9^/L	1.50 (1.26, 1.78)	1.49 (1.26, 1.77)	1.58 (1.37, 1.91)	0.009
Neutrophils, 10^9^/L	5.77 (4.85, 6.87)	5.76 (4.84, 6.86)	6.38 (5.32, 7.38)	<.001
Platelets, 10^9^/L	205.00 (171.00, 239.00)	204.00 (171.00, 238.00)	218.00 (187.50, 253.50)	<.001
LHR	0.96 (0.77, 1.19)	0.95 (0.77, 1.19)	1.04 (0.85, 1.41)	<.001
MHR	0.22 (0.18, 0.28)	0.22 (0.18, 0.28)	0.26 (0.20, 0.34)	<.001
NHR	3.67 (2.98, 4.54)	3.66 (2.97, 4.53)	4.34 (3.29, 5.42)	<.001
PHR	128.92 (103.76, 160.69)	128.57 (103.58, 160.39)	151.05 (114.57, 182.62)	<.001
TC, mmol/L	4.30 (3.87, 4.78)	4.30 (3.86, 4.78)	4.41 (4.04, 4.99)	0.055
TG, mmol/L	1.26 (1.01, 1.59)	1.26 (1.01, 1.59)	1.48 (1.16, 1.86)	<.001
HDL-C, mmol/L	1.57 (1.38, 1.77)	1.57 (1.38, 1.77)	1.51 (1.26, 1.73)	0.014
LDL-C, mmol/L	2.33 (2.04, 2.65)	2.33 (2.04, 2.65)	2.47 (2.12, 2.76)	0.006
Gestational age at delivery	39.00 (38.00, 39.00)	39.00 (38.00, 39.00)	38.00 (38.00, 39.00)	<.001
Gestational diabetes, n(%)				0.002
no	4624 (77.60)	4542 (77.84)	82 (66.13)	
yes	1335 (22.40)	1293 (22.16)	42 (33.87)	
Mode of delivery, n(%)				<.001
Spontaneous delivery	2618 (43.93)	2597 (44.51)	21 (16.94)	
Cesarean section	3341 (56.07)	3238 (55.49)	103 (83.06)	

Data are presented as mean ± SD or median (IQR) for continuous variables and n (%) for categorical variables.

^a^ Cited as mean ± SD. Abbreviations: BMI = body mass index; WBC: White blood cell; LHR: lymphocyte-to-high-density lipoprotein ratio; MHR: monocyte-to-high-density lipoprotein ratio; NHR: neutrophil-to-high-density lipoprotein ratio; PHR: platelet-to-high-density lipoprotein ratio.

### HDL-related inflammation index and PE Risk

In continuous analyses, higher levels of LHR, MHR, NHR, and PHR were significantly associated with an increased risk of PE. After multivariable adjustment, the elevated LHR (RR = 1.99, 95% CI: 1.17–3.42, *P* = 0.012), MHR (RR = 2.62, 95% CI: 1.58–4.35, *P* < 0.001), NHR (RR = 2.87, 95% CI: 1.64–5.02, *P* < 0.001), and PHR (RR = 2.57, 95% CI: 1.48–4.48, *P* < 0.001) remained significantly associated with higher PE risk (Table 2).

**Table 2:** HDL-related inflammatory indices and PE risk.

Variables		Model 1		Model 2	
	n/N (%)	RR (95%CI)	*P*	RR (95%CI)	*P*
**LHR**		2.82 (1.66, 4.77)	<0.001	1.99 (1.17, 3.42)	0.012
**Quartiles of LHR**					
Q1	24/1566 (1.5)	Reference			
Q2	27/1502 (1.8)	1.17 (0.68, 2.05)	0.57	1.08 (0.62, 1.89)	0.788
Q3	24/1410 (1.7)	1.11 (0.63, 1.96)	0.716	0.98 (0.55, 1.74)	0.95
Q4	49/1481 (3.3)	2.16 (1.34, 3.58)	0.002	1.64 (1.00, 2.76)	0.056
*P* for trend			<0.001		0.012
**MHR**		3.60 (2.21, 5.83)	<0.001	2.62 (1.58, 4.35)	<0.001
**Quartiles of MHR**					
Q1	22/1747 (1.3)	Reference			
Q2	17/1263 (1.3)	1.07 (0.56, 2.01)	0.837	0.97 (0.51, 1.83)	0.928
Q3	30/1469 (2.0)	1.62 (0.94, 2.84)	0.085	1.44 (0.83, 2.53)	0.198
Q4	55/1480 (3.7)	2.95 (1.83, 4.94)	<0.001	2.21 (1.34, 3.75)	0.002
*P* for trend			<0.001		<0.001
**NHR**					
**Quartiles of NHR**		4.06 (2.37, 6.94)	<0.001	2.87 (1.64, 5.02)	<0.001
Q1	18/1511 (1.2)	Reference			
Q2	28/1482 (1.9)	1.59 (0.88, 2.92)	0.127	1.49 (0.83, 2.75)	0.186
Q3	26/1487 (1.7)	1.47 (0.81, 2.72)	0.211	1.29 (0.71, 2.39)	0.415
Q4	52/1479 (3.5)	2.95 (1.76, 5.18)	<0.001	2.20 (1.29, 3.93)	0.005
*P* for trend			<0.001		<0.001
**PHR**		3.63 (2.14, 6.16)	<0.001	2.57 (1.48, 4.48)	<0.001
**Quartiles of PHR**					
Q1	20/1491 (1.3)	Reference			
Q2	25/1489 (1.7)	1.25 (0.70, 2.28)	0.454	1.18 (0.66, 2.15)	0.583
Q3	29/1490 (1.9)	1.45 (0.83, 2.60)	0.2	1.27 (0.72, 2.28)	0.418
Q4	50/1489 (3.4)	2.50 (1.51, 4.30)	<0.001	1.86 (1.11, 3.24)	0.023
*P* for trend			<0.001		<0.001

Note: n/N (%): Number of PE a cases/ Total number in the subgroup (Percentage of cases).

Model 1: Crude

Model 2: Adjust for age, gestational weight gain, pre-pregnancy BMI, gestational diabetes, fasting blood glucose, gestational age at the time of blood testing and parity.

Abbreviations: PE = preeclampsia; BMI = body mass index; WBC: White blood cell; LHR: lymphocyte-to-high-density lipoprotein ratio; MHR: monocyte-to-high-density lipoprotein ratio; NHR: neutrophil-to-high-density lipoprotein ratio; PHR: platelet-to-high-density lipoprotein ratio.

In categorical analyses, women in the highest quartile had a higher risk than those in the lowest quartile for MHR (RR = 2.21, 95% CI: 1.34–3.75, *P* = 0.002), NHR (RR = 2.20, 95% CI: 1.29–3.93, *P* = 0.005), and PHR (RR = 1.86, 95% CI: 1.11–3.24, *P* = 0.023), while LHR showed a borderline association (RR = 1.64, 95% CI: 1.00–2.76, *P* = 0.056). All four indices showed significant positive trends with PE risk (all *P* for trend < 0.05, Table 2).

**Fig 2:**
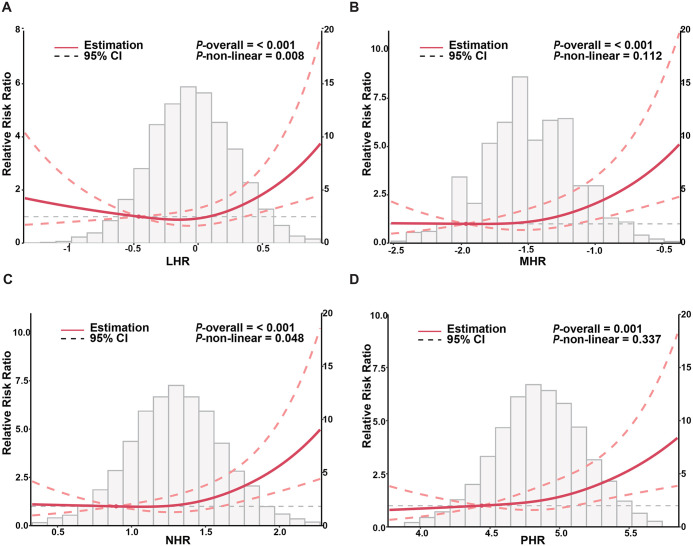
Restricted cubic spline (RCS) curves of four HDL-related inflammatory indices. LHR: lymphocyte-to-HDL cholesterol ratio; MHR: monocyte-to-HDL cholesterol ratio; NHR: neutrophil-to-HDL cholesterol ratio; PHR: platelet-to-HDL cholesterol ratio.

RCS modeling based on AIC minimization selected three knots for all HDL-related inflammation indexs, located approximately at the 5th, 50th, and 95th percentiles of their distributions. The estimated knot values were −0.58, −0.04, and 0.51 for LHR; −2.04, −1.56, and −0.89 for MHR; 0.77, 1.30, and 1.71 for NHR; and 4.31, 4.86, and 5.40 for PHR. LHR and NHR demonstrated statistically significant U-shaped relationships with PE risk (*P* non-linearity = 0.008 and 0.048, respectively), whereas the relationships for MHR and PHR appeared approximately linear (Fig 2). In the sensitivity analysis excluding extreme values below the 1st percentile and above the 99th percentile, all four HDL-related inflammation index (LHR, MHR, NHR, and PHR) demonstrated approximately linear associations with PE risk (S1 Fig). This suggests that the previously observed U-shaped relationship for NHR may be partly driven by extreme observations, while the overall direction of association remained consistent.

### HDL-related inflammation index and PE risk across subgroups

**Fig 3:**
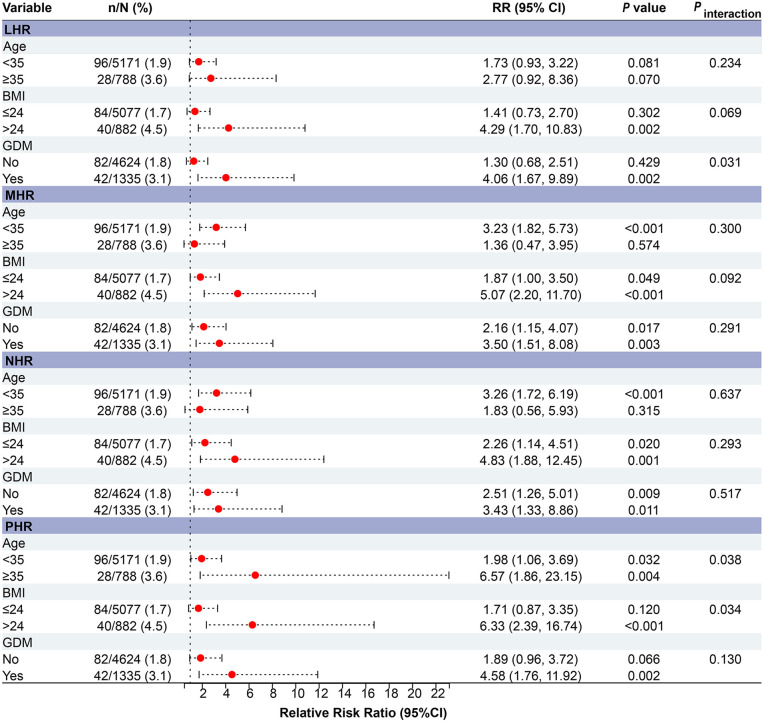
Forest plot of subgroup analyses of HDL-related inflammatory indices and preeclampsia (PE) risk. CI = confidence interval; RR = Relative Risk Ratio; LHR: lymphocyte-to-HDL cholesterol ratio; MHR: monocyte-to-HDL cholesterol ratio; NHR: neutrophil-to-HDL cholesterol ratio; PHR: platelet-to-HDL cholesterol ratio.

In subgroup analyses, the positive associations between HDL-related inflammatory markers and PE were generally consistent across age, pre-pregnancy BMI, and GDM status (Fig 3). Although LHR and PHR appeared more strongly associated with PE risk among women aged ≥ 35 years, women with BMI ≥ 24 kg/m², and women with GDM, these estimates were based on a limited number of PE cases and should be interpreted with caution.

For example, LHR showed a higher estimated risk among women with GDM (RR = 4.06, 95% CI: 1.67–9.89, *P* = 0.002), and the multiplicative interaction reached statistical significance (*P* interaction = 0.031). Similarly, PHR demonstrated stronger estimated associations among women aged ≥ 35 years (RR = 6.57, 95% CI: 1.86–23.15, *P* = 0.003) and among those with elevated BMI (RR = 6.33, 95% CI: 2.39–16.74, *P* < 0.001), accompanied by interaction *P* values for age (*P* interaction = 0.038) and BMI (*P* interaction = 0.034; Fig 3). However, the wide CIs indicate uncertainty, and therefore these associations should be interpreted with caution.

Results of additive interaction analyses are provided in the Supplementary Materials (S1 Table), suggesting potential additive interactions of LHR and MHR on PE risk in women with BMI ≥ 24 kg/m²or GDM, though estimates were imprecise and should be interpreted cautiously. Therefore, these findings should be regarded as exploratory rather than confirmatory.

### Sensitivity analyses

Sensitivity analyses using logistic regression and the PSM modified Poisson regression yielded results generally consistent with the main analysis. Positive associations with incident PE were observed for MHR, NHR, and PHR in both approaches, while the association for LHR was attenuated after PSM (PSM: RR = 1.48, 95% CI: 0.93–2.37, *P* = 0.097), supporting the robustness of the findings (Table 3).

**Table 3:** Sensitivity analyses of associations between HDL-related inflammatory indices and PE risk.

Variables	Logistic regression model^a^		PSM model^b^	
	OR (95%CI)	*P*	RR (95%CI)	*P*
LHR	2.04 (1.18, 3.54)	0.011	1.48 (0.93, 2.37)	0.097
MHR	2.72 (1.62, 4.56)	<0.001	1.85 (1.21, 2.83)	0.004
NHR	2.98 (1.68, 5.29)	<0.001	1.93 (1.20, 3.11)	0.006
PHR	2.66 (1.52, 4.68)	<0.001	1.83 (1.16, 2.88)	0.010

^a^Adjust for age, gestational weight gain, pre-pregnancy BMI, gestational diabetes, fasting blood glucose, gestational age at the time of blood testing and parity.

^b^Crude

Abbreviations: PE = preeclampsia; BMI = body mass index; WBC: White blood cell; LHR: lymphocyte-to-high-density lipoprotein ratio; MHR: monocyte-to-high-density lipoprotein ratio; NHR: neutrophil-to-high-density lipoprotein ratio; PHR: platelet-to-high-density lipoprotein ratio.

### Prediction model performance

**Fig 4:**
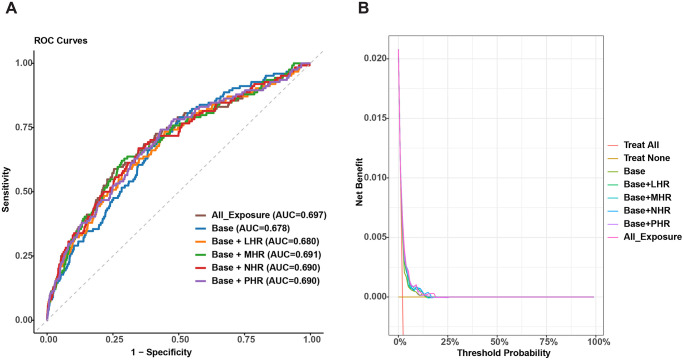
Predictive performance of HDL-related inflammatory indices for PE. **(A)** Receiver operating characteristic (ROC) Curves; **(B)** Decision curve analysis (DCA).

The base model achieved an AUC of 0.678 (95% CI: 0.632–0.725). Inclusion of individual HDL-related inflammation indices modestly improved discrimination: LHR (AUC = 0.680, 95% CI: 0.630–0.731, DeLong test comparing with the base model: *P* = 0.811), MHR (AUC = 0.691, 95% CI: 0.641–0.741, *P* = 0.299), NHR (AUC = 0.690, 95% CI: 0.639–0.740, *P* = 0.318), and PHR (AUC = 0.690, 95% CI: 0.640–0.739, *P* = 0.272). The model including all four indices yielded the highest AUC of 0.697 (95% CI: 0.646–0.748, Fig 4A and S2 Table), but improvement relative to the base model did not reach statistical significance (*P* = 0.140). Calibration evaluation indicated that predicted probabilities were highly consistent with observed risks across all models, with calibration intercepts close to zero and slopes close to one (S2 Fig, S3 Table). DCA showed that the addition of HDL-related inflammatory indices resulted in incremental net benefit over the base model within a narrow threshold probability range, with the all-exposure model demonstrating benefit between 0 and 0.18. Across all models, the maximum net benefit was similar and occurred near a threshold probability approaching zero, consistent with the observed low prevalence of PE in the cohort (Fig 4B, S4 Table).

## Discussion

In this large retrospective cohort of 5,959 women, we observed that four HDL-related inflammatory indices (LHR, MHR, NHR, and PHR) were positively associated with the risk of PE. MHR and PHR showed approximately linear relationships, whereas LHR and NHR demonstrated U-shaped patterns, although sensitivity analyses suggested that these nonlinear associations may be partly influenced by extreme values. Subgroup analyses revealed stronger associations in women with GDM, advanced maternal age, or elevated BMI, suggesting potential effect modification. Sensitivity analyses using logistic regression and PSM confirmed the robustness of our findings. Furthermore, the addition of HDL-related indices resulted in a slight numerical improvement in the AUC of PE risk models, though this incremental gain did not reach statistical significance, suggesting a limited additive value over conventional clinical factors alone.

In this large retrospective cohort, all four HDL-related inflammatory indices, LHR, MHR, NHR, and PHR, were positively associated with PE risk, consistent with the established pathophysiological framework of systemic inflammation and endothelial dysfunction [4–6]. Notably, women with PE exhibited lower HDL-C levels, which may compromise its anti-inflammatory, antioxidative, and vasoprotective functions, a pattern that has been hypothesized to relate to endothelial and placental dysfunction in previous studies [7,10]. Abnormal activation of peripheral immune cells further may further contribute to PE pathogenesis. However, because this study is observational, these associations cannot be interpreted as causal. Early subclinical placental changes may alter lipid metabolism and inflammatory cell activity before clinical diagnosis, meaning that elevated HDL-related indices could also reflect early disease processes rather than contributing factors. Thus, elevated HDL-related indices may reflect a dual pathological process. They may indicate increased proinflammatory activity reflected by higher white blood cells or platelet counts, together with reduced HDL-mediated vascular protection. These patterns may act as markers of disease biology rather than proven mechanistic drivers. This interpretation supports the potential value of these indicators for early risk stratification, although further longitudinal and mechanistic research is needed.

From a biological-hypothesis perspective, each index captures distinct immune-lipid interactions. Elevated LHR reflects lymphocyte imbalance, promoting differentiation toward proinflammatory Th1/Th17 phenotypes and enhancing systemic and placental inflammation [44,45]. Increased MHR, particularly in intermediate monocytes, is associated with proinflammatory cytokine release, oxidative stress, and inflammasome activation, aggravating endothelial and placental injury [19,20,46,47]. Higher NHR indicates neutrophil overactivation, may contributing to endothelial damage through reactive oxygen species, neutrophil extracellular traps (NETs), and proteases [48,49]. Elevated PHR denotes increased platelet activity, which supports vascular remodeling under physiological conditions but promotes thrombosis and vascular dysfunction when excessively activated [50–52]. These prior findings provide plausible mechanisms but do not imply causality in our cohort. Future studies using longitudinal sampling, Mendelian randomization, or interventional approaches are needed to assess temporality and causal direction.

The U-shaped relationships of LHR and NHR highlight the complexity of immune regulation during pregnancy. Both very low and very high LHR levels were associated with increased risk of PE. Low LHR levels may indicate immune suppression or dysfunction, whereas high LHR levels reflect excessive inflammatory activation. Sensitivity analyses excluding extreme values below the 1st percentile and above the 99th percentile showed approximately linear associations, suggesting that these extreme values partly contributed to the observed U-shaped pattern. Extremely low values are likely to reflect rare biological variation rather than measurement error, as measurements underwent standard quality control and their distribution is biologically plausible. Despite the attenuation of the nonlinear pattern, the overall positive association between HDL-related inflammatory indices and PE risk remained consistent.

In the subgroup analyses, we examined potential interactions between inflammatory indices and maternal metabolic factors. Statistical tests identified significant multiplicative interactions between LHR and GDM and between PHR and age and BMI. However, the clinical relevance of these findings remains uncertain because of the limited number of events. The wide CIs around the subgroup risk estimates indicate substantial imprecision, which is likely related to the small sample sizes in stratified analyses. Additive interaction analyses also suggested the possibility of combined effects in metabolically high-risk groups, but these estimates lacked precision. For this reason, the subgroup findings should be interpreted as exploratory rather than confirmatory. They indicate a possible relationship between systemic inflammation and metabolic dysfunction, but further studies in larger populations are needed to validate these observations.

This study has several strengths, including a large sample size, rigorous adjustment for confounders, evaluation of nonlinear relationships, and robust sensitivity analyses incorporating propensity score matching. The study also rigorously assessed the predictive potential of these markers using DCA and calibration plots. Nonetheless, certain limitations should be acknowledged. The single-center, retrospective design may limit generalizability. HDL-related inflammatory indices were measured only once in early pregnancy, precluding assessment of temporal trajectories. Despite extensive covariate adjustment, residual confounding cannot be completely excluded. Specifically, potential confounders such as maternal smoking, socioeconomic status, assisted reproductive technologies, and prior PE were unavailable in our dataset. Subgroup analyses were limited by relatively small sample sizes in certain strata, leading to wide CIs and reduced precision. Validation in larger cohorts is needed to confirm subgroup-specific associations. Although adding HDL-related inflammatory markers slightly improved model discrimination and produced a modest clinical benefit, the improvement was small. These markers may therefore function as supplementary indicators rather than independent predictors. Reverse causation also remains possible. Early placental dysfunction may occur first and then influence lipid profiles and leukocyte counts, rather than the markers contributing to disease development. In addition, this study focused exclusively on late-onset PE, excluding early-onset cases based on evidence that the two subtypes differ in pathophysiological mechanisms and clinical profiles, an approach that reduced etiological heterogeneity and enabled more precise assessment of late-onset specific associations but may limit applicability of the findings to early-onset PE populations.

In conclusion, this study demonstrates that elevated HDL-related inflammatory indices are independently associated with increased risk of PE, underscoring the interplay between inflammation and lipid metabolism during pregnancy. These indices are simple, cost-effective, and readily obtainable from routine laboratory tests, supporting their potential application in early risk stratification and targeted surveillance. Future studies should validate these findings in diverse populations and clinical settings, investigate longitudinal changes during pregnancy, and further elucidate the mechanistic links between lipid metabolism and immune activity. Combining HDL-related indices with other emerging biomarkers (e.g., angiogenic factors or placenta-derived exosomes) may further optimize predictive models and inform precision prevention strategies for PE.

## Supporting information

S1 Table Additive interaction between HDL-related inflammatory indices and PE risk across subgroups of maternal age, pre-pregnancy BMI, and gestational diabetes mellitus (GDM). (DOCX)

S2 Table Predictive performance of models for PE: AUC and DeLong test comparisons. (DOCX)

S3 Table Calibration performance of models for PE. (DOCX)

S4 Table Decision curve analysis of predicted PE Risk. (DOCX)

S1 Fig Sensitivity analysis of RCS curves after exclusion of extreme values. (PDF)

S2 Fig Calibration curves. (PDF)
