# Supplementary material for: Early-pregnancy HDL-related inflammatory indices and risk of preeclampsia: A retrospective cohort study
Source: PLoS One. 2025 Dec 30;20(12):e0339322. doi: 10.1371/journal.pone.0339322 (PMC12753046; doi:10.1371/journal.pone.0339322)
Supplement: S1 Table — (DOCX) [file pone.0339322.s001.docx]

| **Table S1. Additive interaction between HDL-related inflammatory indices and PE risk across subgroups of maternal age, pre-pregnancy BMI, and gestational diabetes mellitus (GDM)** | | | | | | |
| --- | --- | --- | --- | --- | --- | --- |
| Variables | Age | | Pre-pregnancy BMI | | Gestational Diabetes Mellitus | |
|  | RERI (95%CI) | AP (95%CI) | RERI (95%CI) | AP (95%CI) | RERI (95%CI) | AP (95%CI) |
| LHR | 3.46 (-0.44, 13.59) | 0.60 (-0.40, 0.74) | 5.23 (0.79, 14.06) | 0.70 (0.12, 0.82) | 3.43 (0.54, 9.35) | 0.70 (0.08, 0.83) |
| MHR | -1.53 (-3.73, 13.56) | -1.24 (-38.79, 6.18) | 23.59 (12.73, 225.01) | 0.76 (-0.05, 0.83) | 6.14 (3.95, 71.36) | 0.62 (-1.63, 0.97) |
| NHR | 1.43 (-11.91, 11.40) | 0.23 (-0.47, 0.55) | 1.34 (-2.02, 7.56) | 0.39 (0.02, 0.82) | 0.50 (-2.97, 4.95) | 0.18 (-0.30, 0.68) |
| PHR | -0.92 (-3.38, 2.28) | -94.03 (-53694.72, 36373.83) | -0.72 (-3.36, 2.65) | -34.93 (-10526.55, 8870.77) | -0.85 (-6.94, 9.59) | -10.60 (-2187.79, 1687.70) |
| Abbreviations: RERI = Relative Excess Risk of Interaction; AP = Attributable Proportion of Interaction; CI = Confidence Interval; LHR: lymphocyte-to-high-density lipoprotein cholesterol ratio; MHR: monocyte-to-high-density lipoprotein cholesterol ratio; NHR: neutrophil-to-high-density lipoprotein cholesterol ratio; PHR: platelet-to-high-density lipoprotein cholesterol ratio. | | | | | | |
