# Supplementary material for: Early-pregnancy HDL-related inflammatory indices and risk of preeclampsia: A retrospective cohort study
Source: PLoS One. 2025 Dec 30;20(12):e0339322. doi: 10.1371/journal.pone.0339322 (PMC12753046; doi:10.1371/journal.pone.0339322)
Supplement: S2 Table — (DOCX) [file pone.0339322.s002.docx]

| **Table S2. Predictive performance of models for PE: AUC and DeLong test comparisons** | | |
| --- | --- | --- |
| Model | AUC (95% CI) | *P* value vs Base (DeLong test) |
| Base | 0.678 (0.632–0.725) | – |
| LHR | 0.680 (0.630–0.731) | 0.811 |
| MHR | 0.691 (0.641–0.741) | 0.299 |
| NHR | 0.690 (0.639–0.740) | 0.318 |
| PHR | 0.690 (0.640–0.739) | 0.272 |
| All_Exposure | 0.697 (0.646–0.748) | 0.140 |
| Abbreviations: AUC: Receiver Operating Characteristic Curves CI: Confidence Interval; LHR: lymphocyte-to-high-density lipoprotein cholesterol ratio; MHR: monocyte-to-high-density lipoprotein cholesterol ratio; NHR: neutrophil-to-high-density lipoprotein cholesterol ratio; PHR: platelet-to-high-density lipoprotein cholesterol ratio. | | |
