# Supplementary material for: Early-pregnancy HDL-related inflammatory indices and risk of preeclampsia: A retrospective cohort study
Source: PLoS One. 2025 Dec 30;20(12):e0339322. doi: 10.1371/journal.pone.0339322 (PMC12753046; doi:10.1371/journal.pone.0339322)
Supplement: S3 Table — (DOCX) [file pone.0339322.s003.docx]

| **Table S3. Calibration performance of models for PE** | | | |
| --- | --- | --- | --- |
| Model | Calibration Intercept | Slope | Mean Absolute Error |
| Base | -6.4E-12 | 1 | 0.002 |
| LHR | -9.1E-12 | 1 | 0.002 |
| MHR | -2.3E-11 | 1 | 0.002 |
| NHR | -1.9E-11 | 1 | 0.002 |
| PHR | -2E-11 | 1 | 0.002 |
| All_Exposure | -3.4E-11 | 1 | 0.002 |
| Abbreviations: LHR: lymphocyte-to-high-density lipoprotein cholesterol ratio; MHR: monocyte-to-high-density lipoprotein cholesterol ratio; NHR: neutrophil-to-high-density lipoprotein cholesterol ratio; PHR: platelet-to-high-density lipoprotein cholesterol ratio. | | | |
