# Supplementary material for: Early-pregnancy HDL-related inflammatory indices and risk of preeclampsia: A retrospective cohort study
Source: PLoS One. 2025 Dec 30;20(12):e0339322. doi: 10.1371/journal.pone.0339322 (PMC12753046; doi:10.1371/journal.pone.0339322)
Supplement: S4 Table — (DOCX) [file pone.0339322.s004.docx]

| **Table S4. Decision Curve Analysis of Predicted PE Risk** | | | |
| --- | --- | --- | --- |
| **Model** | **Max Net Benefit** | **Threshold at Max NB** | **Risk Threshold Range with Positive NB** |
| Base | 0.0208 | 0 | 0 – 0.13 |
| LHR | 0.0208 | 0 | 0 – 0.14 |
| MHR | 0.0208 | 0 | 0 – 0.14 |
| NHR | 0.0208 | 0 | 0 – 0.16 |
| PHR | 0.0208 | 0 | 0 – 0.16 |
| All_Exposure | 0.0208 | 0 | 0 – 0.18 |
| Abbreviations: LHR: lymphocyte-to-high-density lipoprotein cholesterol ratio; MHR: monocyte-to-high-density lipoprotein cholesterol ratio; NHR: neutrophil-to-high-density lipoprotein cholesterol ratio; PHR: platelet-to-high-density lipoprotein cholesterol ratio. | | | |
