# Supplementary figures and images for: Early-pregnancy HDL-related inflammatory indices and risk of preeclampsia: A retrospective cohort study
Source: PLoS One. 2025 Dec 30;20(12):e0339322. doi: 10.1371/journal.pone.0339322 (PMC12753046; doi:10.1371/journal.pone.0339322)

A

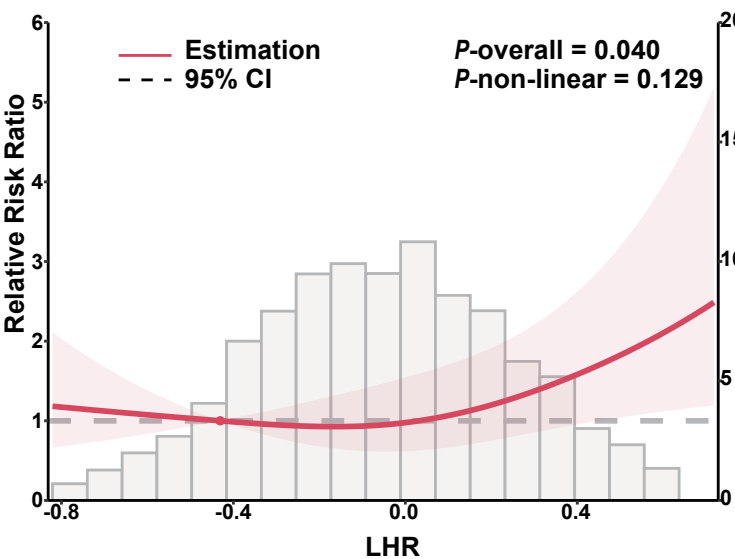

B

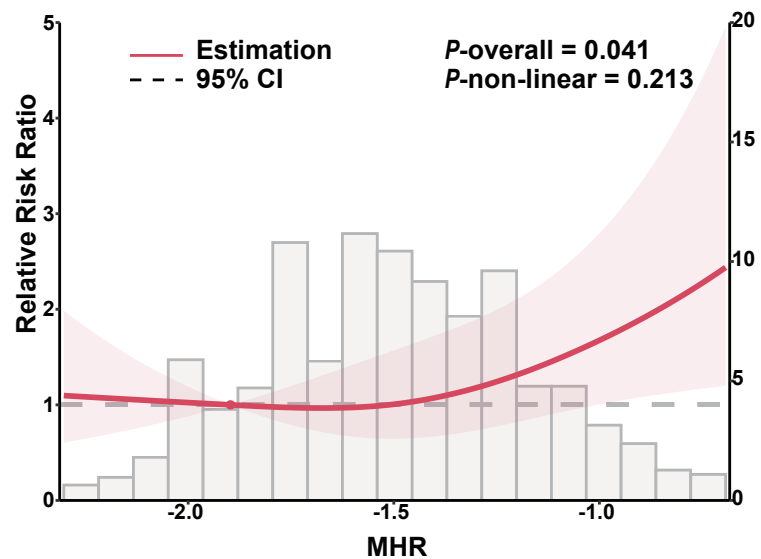

C

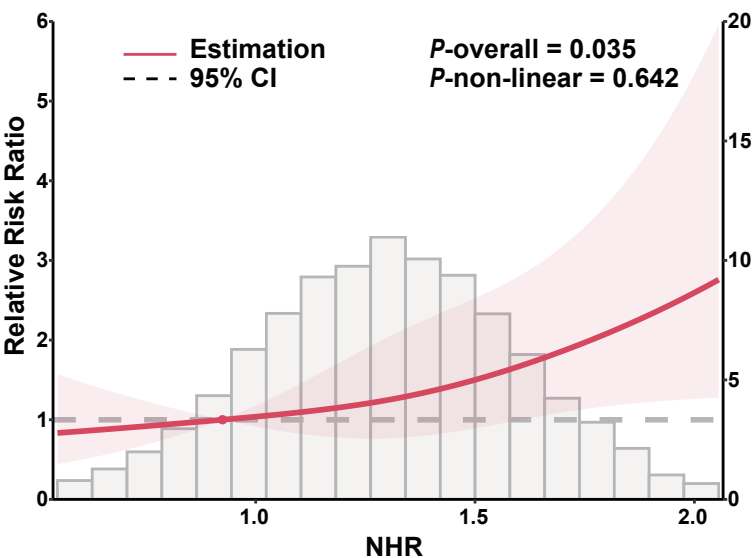

D

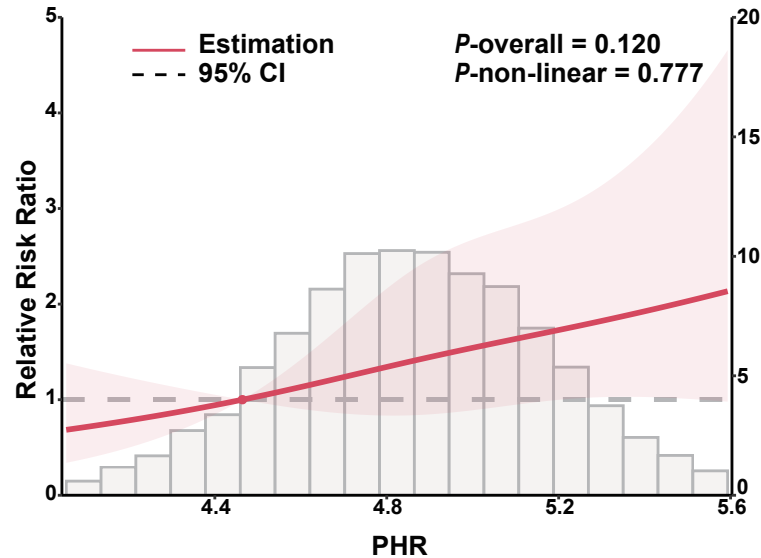

Supplement: S1 Fig — (PDF) [file pone.0339322.s005.pdf]

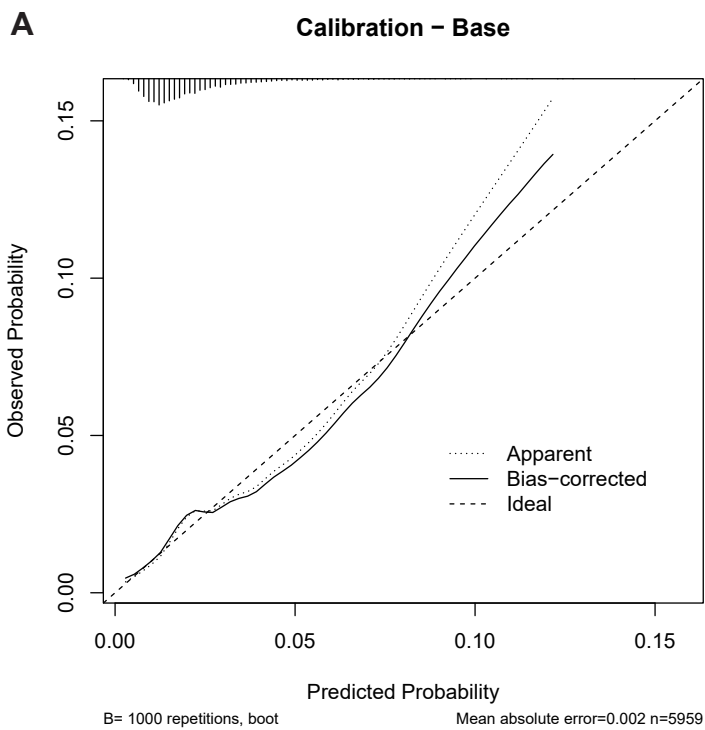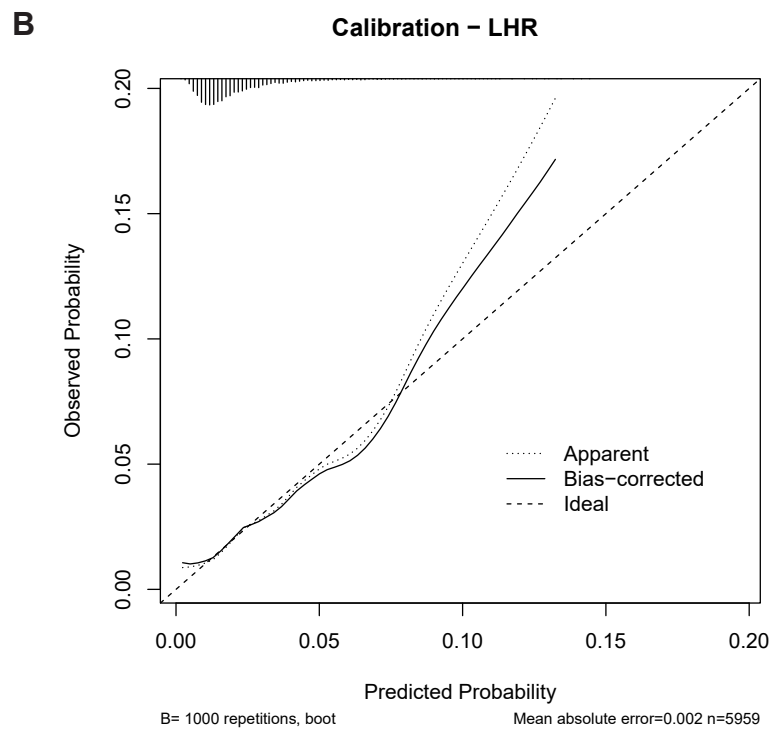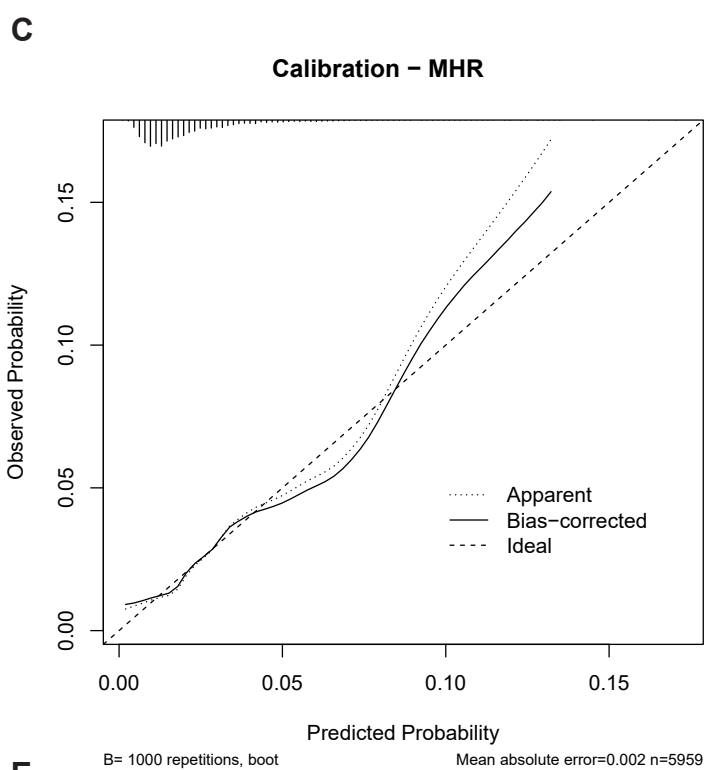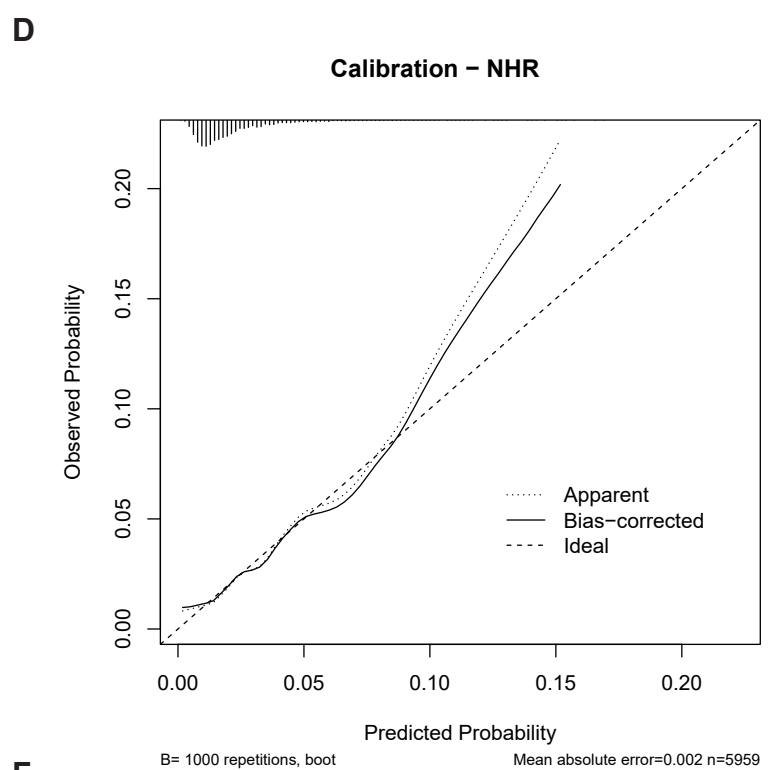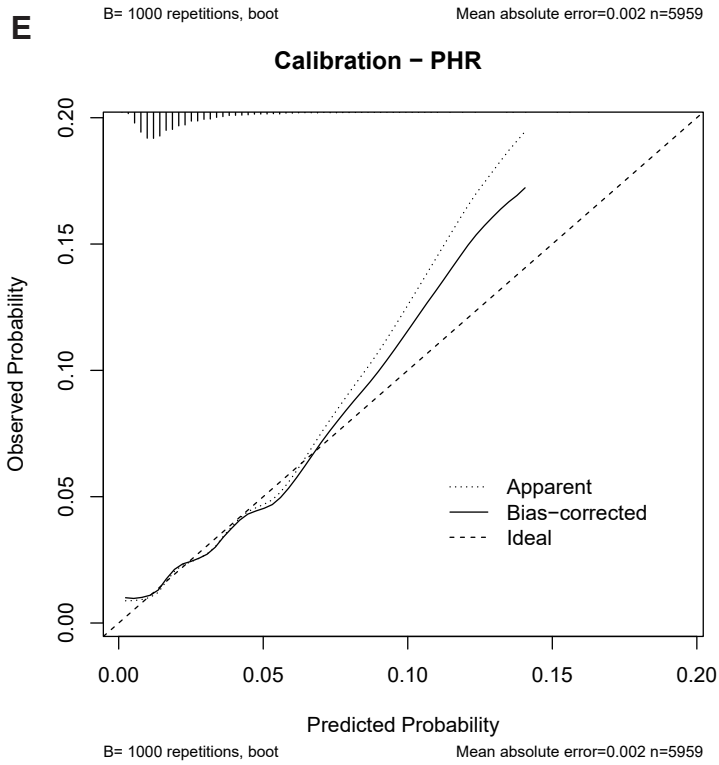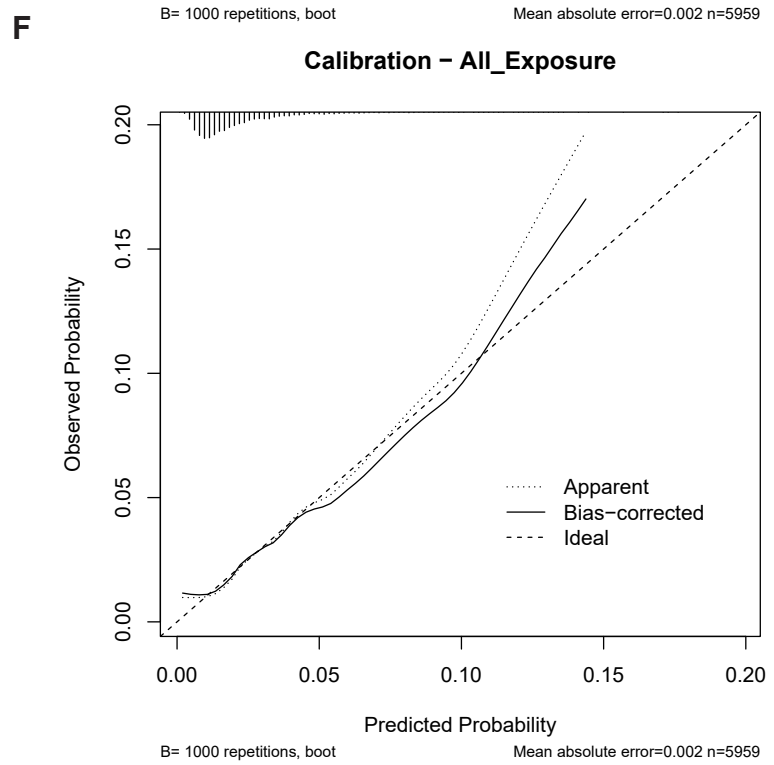

Supplement: S2 Fig — (PDF) [file pone.0339322.s006.pdf]
